# Mining Public Mass Spectrometry Data to Characterize the Diversity and Ubiquity of *P. aeruginosa* Specialized Metabolites

**DOI:** 10.3390/metabo10110445

**Published:** 2020-11-05

**Authors:** Andrew C. Lybbert, Justin L. Williams, Ruma Raghuvanshi, A. Daniel Jones, Robert A. Quinn

**Affiliations:** 1 Department of Biochemistry and Molecular Biology, Michigan State University, East Lansing, MI 48823, USA; andrew.lybb@gmail.com (A.C.L.); williamsjustin5662@gmail.com (J.L.W.); raghuvan@msu.edu (R.R.); jonesar4@msu.edu (A.D.J.); 2 Department of Biology, University of Arkansas at Pine Bluff, Pine Bluff, AR 71601, USA

**Keywords:** *Pseudomonas aeruginosa*, cystic fibrosis, specialized metabolites, GNPS, quinolone

## Abstract

*Pseudomonas aeruginosa* is a ubiquitous environmental bacterium that causes chronic infections of burn wounds and in the lungs of cystic fibrosis (CF) patients. Vital to its infection is a myriad of specialized metabolites that serve a variety of biological roles including quorum sensing, metal chelation and inhibition of other competing bacteria. This study employed newly available algorithms for searching individual tandem mass (MS/MS) spectra against the publicly available Global Natural Product Social Molecular Networking (GNPS) database to identify the chemical diversity of these compounds and their presence in environmental, laboratory and clinical samples. For initial characterization, the metabolomes of eight clinical isolates of *P. aeruginosa* were analyzed using liquid chromatography-tandem mass spectrometry (LC-MS/MS) and uploaded to GNPS for spectral searching. Quinolones, rhamnolipids, phenazines and siderophores were identified and characterized; including the discovery of modified forms of the iron chelator pyochelin. Quinolones were highly diverse with the three base forms *Pseudomonas* quinolone signal 2-heptyl-3-hydroxy-4(*1H*)-quinolone (PQS), 4-heptyl-4(*1H*)-quinolone (HHQ) and 2-heptyl-4-quinolone-*N*-oxide (HQNO) having extensive variation in the length of their acyl chain from as small as 3 carbons to as large as 17. Rhamnolipids were limited to either one or two sugars with a limited set of fatty acyl chains, but the base lipid form without the rhamnose was also detected. These specialized metabolites were identified from diverse sources including ant-fungal mutualist dens, soil, plants, human teeth, feces, various lung mucus samples and cultured laboratory isolates. Their prevalence in fecal samples was particularly notable as *P. aeruginosa* is not known as a common colonizer of the human gut. The chemical diversity of the compounds identified, particularly the quinolones, demonstrates a broad spectrum of chemical properties within these the metabolite groups with likely significant impacts on their biological functions. Mining public data with GNPS enables a new approach to characterize the chemical diversity of biological organisms, which includes enabling the discovery of new chemistry from pathogenic bacteria.

## 1. Introduction

Mass spectrometers are highly advanced instruments able to accurately measure the mass to charge ratio of a variety of analytes. Historically, these instruments have been used in isolation, with few platforms for easily sharing and cross comparing data between laboratories and across instrument types. Recent advances in the development of publicly available mass spectrometry databases and searching algorithms, such as the Global Natural Products Social Molecular Networking Database [1], Metabolomics Workbench [2], molecular networking [3] and Mass Spectrometry Search Tool (MASST) [4], have enabled comparison of data generated from a wide variety of sample types analyzed using different instruments through MS/MS spectral alignment scoring and molecular network visualization. While limitations exist in the ability to compare MS/MS spectra generated on different instruments with different fragmentation parameters and analytical approaches, there is great potential to mine the immense data available on public repositories for biological insights into untargeted metabolomics experiments regardless of the technical differences between analytical platforms. The ability to mine biological information from publicly available mass spectrometry data is akin to the early stages of genomics, where algorithms such as the Basic Local Alignment Search Tool (BLAST) [5] enabled cross comparison of nucleic acid sequences and the explosion of the field of bioinformatics.

Many bacterial pathogens produce small molecule specialized metabolites that contribute to their colonization and persistence in the host environment. *Pseudomonas aeruginosa* is a particularly good example, as it produces a myriad of small molecule virulence factors known to have effects on host cells and other bacteria during chronic infections. This bacterium is one of the main opportunistic pathogens that colonizes the nutrient rich environment of the CF lung, where these compounds can be detected in clinical mucus samples [6,7]. Most attention has been played to specific chemical forms of these molecules, such as the *Pseudomonas* quinolone signal (2-heptyl-3-hydroxy-4-quinolone, PQS C-7), but there are many other chemical variants [8], and we have little knowledge of their diversity and presence in different environments. The extensive publicly accessible data on the *P. aeruginosa* specialized metabolome in particular, enables a bioinformatic approach to characterization of the bacterium’s specialized metabolite diversity and ubiquity.

This study characterizes the chemical diversity of four specialized metabolite groups produced by clinical isolates of *P. aeruginosa*: quinolones, rhamnolipids, phenazines and siderophores. We used molecular networking [3] and the spectral search algorithm MASST [4] to describe the chemical diversity of these compounds, which is primarily driven by differential length of fatty acyl chains on the quinolone molecular group. This method also enabled discovery of previously undescribed forms of pyochelin and an understanding of the ubiquity of these compounds in the environment, which includes environmental, host associated and laboratory environments.

## 2. Results

Molecular networks were created from the combined metabolome of the clinical isolates and their three different extraction methods. Of the 1490 metabolites not detected in blanks (samples comprised of the extraction solvent only without any sample added), 49.7% were found in all three solvents. Methanol extracts alone detected the highest number of unique metabolites (15.7%) followed by ethyl acetate:methanol (10.7%). Only 2.2% of molecules were exclusively detected in both ethyl acetate:methanol and ethanol, indicating these two solvents had the least overlap of metabolite diversity.

### 2.1. Quinolone Diversity

**Figure 1:**
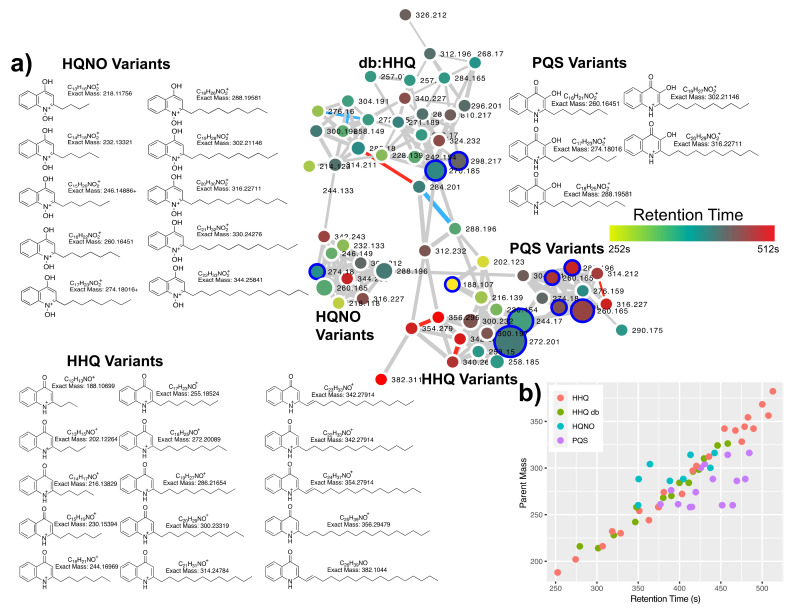
Quinolone diversity from *P. aeruginosa* clinical isolates. (**a**) Molecular network and quinolone structures for compounds identified in this study. The network nodes are colored by retention time according to the scale and sized by the number of spectra in the dataset. Edge width in the network is sized by the cosine score. Red edges represent those of a desaturation (Δ *m/z* = 2) and blue edges represent two desaturations (Δ *m/z* = 4). (**b**) Scatterplot of the parent mass and retention time in seconds of all quinolones identified. Points are colored by type of base quinolone structure as described in the text.

Using the classical networking approach and after removal of hybrid spectra there were 62 nodes connected in the quinolone molecular network with 6 hits in the GNPS libraries identifying them as belonging to the quinolones. There were four different discernable subnetworks within this quinolone molecular family (Figure 1), three of these representing a different base quinolone ring structure of either the HHQ (*m/z* 244.170, C_16_H_22_NO), HQNO (*m/z* 260.165, C_16_H_22_NO_2_) or PQS (*m/z* 260.165, C_16_H_22_NO_2_) type. The fourth subnetwork in this molecular family represented quinolones of the HHQ type, but with an unsaturated C-C bond between the α- and β-carbon of the fatty acyl chain, which produces another fragment ion of *m/z* 184.08, as described in [9] (Figure S1). The MS/MS patterns of the HHQ, PQS and desaturated fatty acyl quinolones are different, and were found to match those as described in [9] (Figure 1). The HHQ and HQNO type quinolones produce identical MS/MS patterns, but have different parent masses due to the different quinolone ring structures. Conversely, PQS type quinolones have the same molecular formulae as those of the HQNO type but result in different MS/MS patterns. The quinolones showed remarkable chemical diversity, primarily driven by variation in the length of the fatty acyl chain. There were quinolones detected as small as 188.107 Da and as large as 388.311 Da, corresponding to fatty acyl chain lengths of three carbons and 17 carbons, respectively, and a retention time difference of 260 s. Both of these extreme quinolones belonged to the HHQ type and some of the longest fatty acids (FAs) on HHQ quinolones were those containing a double bond. HQNO variants were as small as a 5-carbon fatty acyl chain (*m/z* 218.118, Figure 1) and as large as 13 carbons (*m/z* 344.259). The PQS group showed less molecular diversity, with a 7-carbon form being the smallest and an 11-carbon fatty acid as the largest. Using the feature-based molecularl networking (FBMN) method, the most abundant quinolones in the dataset were HHQ, NHQ and PQS in that order (Figure S2).

Quinolone diversity can also arise from desaturation of the fatty acyl chain. To investigate this, we analyzed all cosine connections in the quinolone network with a Δ *m/z* of 2 or 4 representing one or two desaturations. Four pairs of single desaturations were detected and three double desaturations Figure 1. There were no connected nodes in the network with a delta *m/z* of 6 or 8, indicating that two acyl chain desaturations were the most detected in the molecular family.

### 2.2. Rhamnolipid Diversity

**Figure 2:**
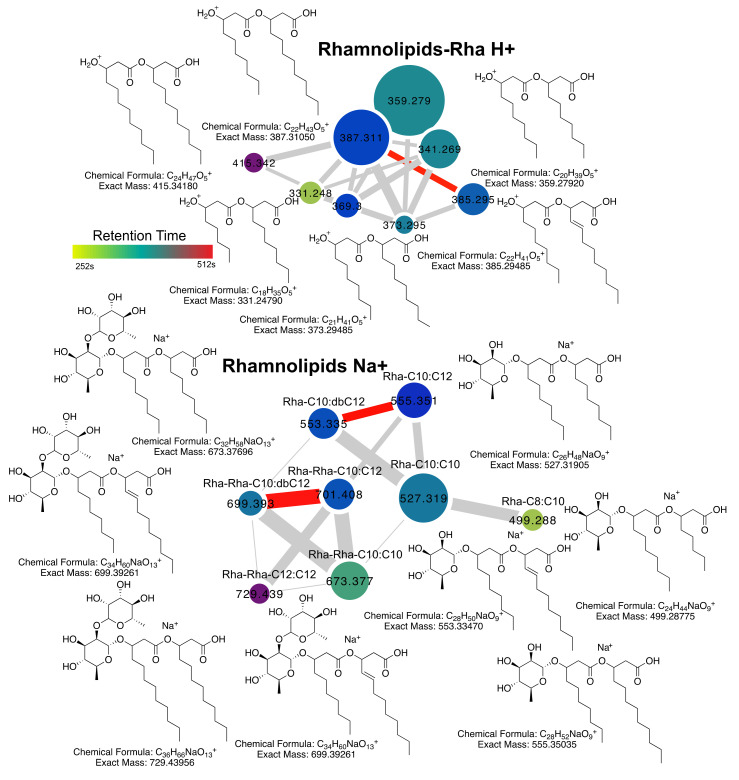
Rhamnolipid diversity from *P. aeruginosa* clinical isolates. Molecular network and rhamnolipid structures for compounds identified in this study. The network nodes are colored by retention time according to the scale and sized by the number of spectra in the dataset. Edge width in the network is sized by the cosine score. Red edges represent those of a desaturation (Δ *m/z* = 2). The position of the FA double bond is unknown.

There were two molecular families in the MS/MS network that contained known rhamnolipids. One that contained the precursor metabolites with no rhamnose 3-(3-hydroxyalkanoyloxy)alkanoic acids (HAAs) [10] connected to H^+^ and NH_4_^+^ adducts of sugar-containing rhamnolipids and a separate network of rhamnolipid Na^+^ adducts. For this study, we analyzed HAA in their H^+^ adduct forms and the Na^+^ adducts of the rhamnoside forms. There were eight HAAs and eight rhamnoside forms (four monorhamnose and four dirhamnose) detected in the cultured isolates (Figure 2). Only C_8_ and C_10_ forms of the rhamnolipid FAs were identified and these were present in the HAA, Rha- and Rha-Rha- forms. A single desaturation on the second rhamnolipid fatty acyl-chain was identified contributing to further chemical diversity of these compounds (Figure 2).

### 2.3. Phenazine Diversity

Phenazines did not network together in a single molecular family, instead, they were connected to other small molecules or found as singletons. Phenazines detected in the data included 1-hydroxyphenazine, pyocyanin, phenazine-1-carboxylic acid, and the lesser known phenazine-1,6-dicarboxylic acid (*m/z* 269.0562).

### 2.4. Pyochelin Diversity

**Figure 3:**
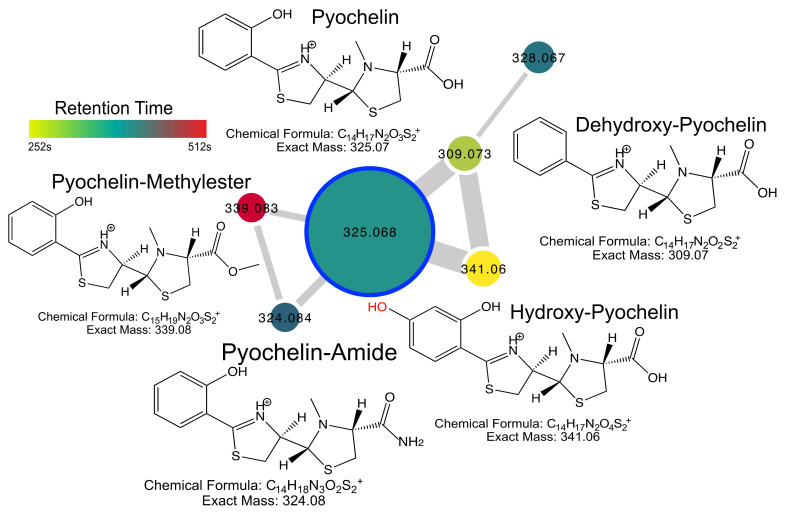
Pyochelin diversity detected in clinical isolates of *P. aeruginosa* culture extracts. Structures, chemical formulas and exact masses of known and putative compounds are shown. The network nodes are colored by retention time according to the scale and sized by the number of spectra in the dataset. Edge width in the network is sized by the cosine score. Note that the hydroxyl highlighted in red is at an unknown position on the aromatic ring.

The iron siderophore pyochelin was detected in a network of six nodes, including pyochelin itself and its known methyl ester form [11], both of which were identified in the GNPS libraries. MS/MS analysis of the other four nodes led to the discovery of three putatively modified forms of the molecule. This included hydroxy-pyochelin, which has a hydroxyl group added to the aromatic ring at an unknown location, dehydroxypyochelin, where the aromatic ring lacks a hydroxyl group, and pyochelin amide, where the carboxylic acid group is replaced by an amide (Figure 3). These unique metabolites have both different retention times and MS/MS patterns than pyochelin supporting their existence as unique molecules (Figures S3–S8).

The diversity of pyochelin was further explored using the MASST molecular networking feature, where all files with hits from a MASST search can be networked together for further analysis (results link in methods). This network of 1338 files that had a hit to pyochelin from our original clinical isolate data resulted in a pyochelin MASST network that reflected further diversity of these siderophores. The hydroxy-pyochelin, dehydroxy-pyochelin and amide forms were all identified in this network with highly similar MS/MS patterns to those described from the clinical cultures, including the diagnostic fragments (Figure S4), which further verifies the existence of the three novel molecules. The MASST network revealed other previously unidentified forms of pyochelin molecules, including a decarboxylated form (*m/z* 281.077, clusterID 732056) and a further hydroxylated form of the methyl-ester (*m/z* 355.078, clusterID 1260530), the putative structures of these compound remain unknown, however.

### 2.5. Ubiquity of P. aeruginosa Specialized Metabolites

**Figure 4:**
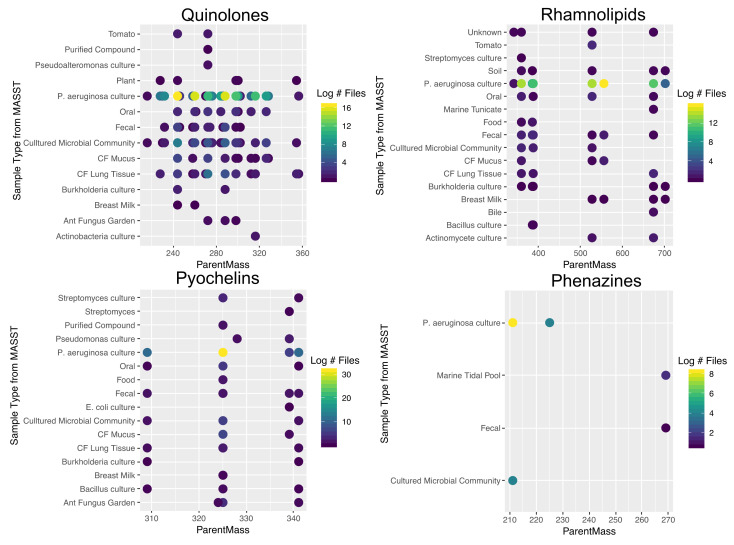
Presence of *P. aeruginosa* specialized metabolites in different sample types on GNPS after MASST searching. Each point represents a unique MS/MS spectrum searched with MASST and the points are colored by the log_10_ number of files that the compounds was detected in within that sample type.

MASST searching [4] was used to identify *P. aeruginosa* specialized metabolites in publicly available metabolomics data on GNPS. All known quinolones in the molecular network (from the feature-based molecular networking) were searched including those of the PQS, HQNO, and HHQ type. Only five nodes had no hits in the GNPS database, three of the HHQ type and two of the PQS type. The distribution of parent masses in GNPS studies showed that particular parent masses were more prevalent than others, specifically, HHQ (*m/z* 244.1697) and HQNO/PQS-C9 (*m/z* 288.1958) being the most common masses (Figure S2). These most prevalent quinolones were identified in cultures of *P. aeruginosa* and mixed microbial cultures as expected, as well as CF lung tissue and mucus clinical samples. There was no discernable difference in the quinolone structures present between those detected in bacterial cultures and in these clinical samples, except that the very small quinolones were not found in clinical samples. Other human samples included those of oral origin (including ground teeth), breast milk and feces, the latter of which showed a diverse set of quinolones, including HHQ, but in few total samples (Figure 4). These molecules were also detected in environmental samples including an ant fungus garden from *Trachymyrmex septentrionalis* and various plants. Other bacterial cultures also contained quinolones including *Burkholderia* spp., a bacterium known to produce these compounds, but also *Pseudoalteromonas* and Actinobacteria. Overall, the MASST searching revealed diverse environmental and human sources for quinolones, many of which are not known to be common sources of the bacterium that produces them.

Rhamnolipids exhibited greater ubiquity in sample presence, being found in many of the same sample types as the quinolones but also in soil, marine tunicates, food samples, and cultures of other bacterial species including *Streptomyces* spp. and Actinomycetes. There was not any trend in particular rhamnolipids being found in particular samples, but it was notable that the HAAs were equally as ubiquitous as the rhamnose forms. Interestingly, the rhamnolipids were not found in the ant fungus garden samples that had many quinolones and pyochelins present.

Pyochelins were also present in diverse sample types similar to the other specialized metabolites, of note, was the prevalence of the novel pyochelins described in this study in many of the same samples as pyochelin itself, further supporting the true existence of these molecules.

Phenazines were not as ubiquitous as the other *P. aeruginosa* metabolites searched with MASST, being found in only four different sample types. This also included stool, where all four molecular families were found and a sample from a marine tidal pool. Pyocyanin was by far the most prevalent of the phenazines as the others were found in fewer samples. This result likely reflects the low number of primary fragments produced by the phenazines with our, and most, MS/MS parameters. Hydroxyphenazine only produces two primary fragments, making many of the MASST searches with a minimum peak match of 3 or four unlikely to detect these compounds. This may be a limitation of the MASST approach as smaller metabolites produce fewer MS/MS fragments making successful searches more challenging.

## 3. Discussion

This study investigated the molecular diversity of *P. aeruginosa* specialized metabolites by searching publicly available data in the GNPS MS/MS database. An initial screen of eight clinical isolates identified the diversity of these compounds detected from different solvent extractions. Subsequent searching of these MS/MS spectra for relatives in the GNPS database expanded the known diversity including the discovery of novel compounds. Thus, we demonstrate how mining of public data with newly available MS/MS search algorithms, such as MASST, can enable a new form of molecular discovery where the putative existence of a compound by its presence in a public dataset can be identified. Simultaneously, one can obtain information about the type of samples that previously known and putative metabolites are found in. This approach could greatly expand the search for natural products as one can identify the potential existence of a compound of interest and then attempt to isolate and structurally characterize it from samples types where it is found.

Particularly notable chemical diversity was found within the *P. aeruginosa* quinolone group. The heptyl- and nonyl-quinolone forms were the most prevalent in both the clinical isolates and the GNPS public data, but quinolones with as small as a 3-carbon FA chain and as large as 17-carbons were also produced by the clinical isolates and identified in GNPS. Interestingly, these extreme quinolone forms were only identified with the HHQ backbone ring structure, indicating the other quinolone forms, PQS and HQNO, may not be created in this manner enzymatically. Nguyen et al. [12] also characterized quinolones and other natural products from pseudomonads and reported some of the same structures identified here, as well as novel forms with alterations on the quinolone rings that we did not identify. However, this study more broadly focused on the *Pseudomonas* genus and did not describe the extreme forms of the quinolones found here with the very short and long FA tails. FAs added to culture media are known to increase PQS production [13] and the structure of quinolones are believed to be derived directly from FAs available in the cellular pool [14,15], but whether or not these unusually short or unusually long quinolones are synthesized *de novo* or captured from the FA pool is unknown. Regardless, the variation in the chain length of these compounds will have significant effects on their biochemistry and functions. The retention time difference between these two extreme quinolones was 260 s showing the strong effect of the FA chain length on the quinolone hydrophobicity. Thus, the length of the FA chain could have a significant effect on the molecules’ diffusive properties during infections and in the environment. Importantly, Depke et al. [16] recently showed that the metabolite profiles of *P. aeruginosa* can predict virulence in model systems, further highlighting the importance of small chemical variations of specialized metabolites, such as those described here, as important for virulence. The different FA chain lengths may induce quorum sensing signaling differently depending on the environment. HHQ, for example, is known to function as a quorum sensing metabolite in *P. aeruginosa* and is excreted into the aqueous environment. The large 17-carbon FA form of HHQ is not likely to diffuse well in an aqueous environment, perhaps making it more functional in the biofilm over shorter distances or in an environment rich in lipids. In contrast, shorter more hydrophilic quinolones may have far reaching quorum sensing effects, able to reach other cells in an aqueous environment farther from that which produced it. The effects of the FA chain length on quinolone function deserves further research to understand how these compounds affect the quorum sensing properties of these molecules.

The three most abundant quinolones from the clinical *P. aeruginosa* dataset were HHQ, NHQ and PQS. Interestingly, in GNPS the three most abundant compounds were HHQ, PQS-C9 and PQS-C7, indicating that the PQS-type quinolone may be more ubiquitous. Detection of these compounds in clinical samples has diagnostic implications, and from our study, the most common quinolones in CF lung or mucus samples were HHQ and NHQ. Thus, these compounds may serve as the most likely quinolones found in these types of samples, with PQS being far less prevalent and not associated with any CF mucus sample from our MASST searching. However, these quinolones were found in cultures from other bacteria, including *Burkholderia* spp., *Pseudoaltereomonas* spp., and Actinobacteria. *Burkholderia* and *Pseudoalteromonas* are known to produce quinolones [17,18], making these compounds alone not sufficient for identification of the bacterium in clinical samples. Pyochelins and rhamnolipids were also identified in *Burkholderia*, but not phenazines. Combinations of metabolite detection from these different families may serve as better diagnostic markers of infection.

The MASST search results demonstrate the broad niche occupancy of *P. aeruginosa* and its metabolites. The compounds of interest were identified in plant, human, microbial and environmental sample types. The quinolones were found in a number of unexpected sample types including feces, breast milk and an ant fungus garden. Nguyen et al. [12], found quinolones highly associated with human samples, thus, our MASST-based searching represents a computational method to expand the knowledge of chemical ubiquity beyond that of culturing individual isolates from diverse sources. The finding of quinolones in a number of fecal samples is somewhat unexpected, as the bacterium is not normally considered an infectious agent of the human gastrointestinal tract, though it has been reported previously [19]. Supporting its presence in feces is the finding of pyochelin, a phenazine and rhamnolipids in the same datasets. This result indicates that LC-MS/MS based detection of these compounds may be a useful method for screening fecal samples for *P. aeruginosa* infection, much as it has been for CF mucus samples [6,20,21], as this can be done rapidly within hours of sample collection [22]. There were few instances where a molecular family of *P. aeruginosa* metabolites was found in only a single sample type, indicating that these compounds generally are produced together across a wide variety of infection sites and environments. Pyochelin, however, was uniquely found in *Streptomyces* cultures and food samples, indicating it may be produced by this environmental bacterium, and accordingly, the biosynthetic genes for its production are present in *Streptomyces* spp. [23]. The detection of rhamnolipids in food, soil, a marine tunicate, and bile samples suggests that these compounds may be more ubiquitous than the other *P. aeruginosa* molecular families. These surfactants are known to be produced by a broad range of pseudomonads [24], possibly explaining their more widespread detection here. The MASST searching performed in this study support the environmental ubiquity of *P. aeruginosa* and indicates that searching for its metabolite production in clinical samples may serve as a useful diagnostic tool, though their detection alone cannot specifically identify *P. aeruginosa*, as other bacteria are found to produce these compounds.

One of the most interesting findings from our exploration of *P. aeruginosa* metabolite diversity was the detection of unique forms of pyochelin, that to our knowledge, have not been previously described. Pyochelin is known to exist in enantiomeric forms [25], and as a methyl ester [26,27]. Molecular networking of the clinical isolate data revealed a molecular family of pyochelins that included pyochelin itself, the methyl-ester and three other unknown masses. These molecules had different retention times from pyochelin, indicating they were not adducts or in source fragments. Detailed MS/MS analysis was able to discern the putative structure of these compounds, though they will need to be validated by other analytical methods, which are outside of the scope of this study. These new compounds are a pyochelin amide, hydroxypyochelin and dehydroxypyochelin. How these compounds were produced enzymatically is unknown, but the dehydroxypyochelin form is particularly interesting because it is missing a hydroxyl on the aromatic ring domain. It is possible that benzoate, which is structurally similar to salicylate but missing the aromatic hydroxyl, could be the precursor due to enzyme promiscuity of PchD which activates salicylate, or other enzymes are involved after synthesis of pyochelin [28]. Similarly, the hydroxypyochelin form, where an additional hydroxyl is added to the aromatic ring may originate from a dihydroxylated precursor such as 3,4-dihydroxybenzoate. However, the MS/MS patterns from our data did not provide definitive determination of the location of the second hydroxyl. The amide is also an interesting additional structure as the PchF mediated release of pyochelin after synthesis is believed to be a hydrolytic process from the thioesterase domain producing the carboxylic acid seen in the parent compound pyochelin [28]. There may be another mechanism of release of the pyochelin structure from the non-ribosomal peptide synthase multi-domain enzyme responsible for pyochelin synthesis, or this too represents a post-processing of the molecule after release. Though these structures are only putative, they represent greater molecular diversity of *P. aeruginosa* siderophores than previously thought. Their presence in GNPS through MS/MS searching further supports their existence. All three novel compounds were identified in other MS/MS datasets from diverse sources. Pyochelin diversity may be even greater than that identified in our clinical isolates, as the molecular networking feature of the MASST search algorithm identified other molecules with highly similar but modified MS/MS patterns to pyochelin; including a decarboxylated form and a further hydroxylated form of the methyl-ester. These compounds are only putative, as MASST provides only MS/MS-based evidence of their existence without retention time information. Nevertheless, this study demonstrates the potential for molecular discovery through MASST searching and the ability to putatively identify novel compounds from some of the best studied producers of bacterial natural products. Though out of the scope of this current study, it is of utmost importance to subsequently identify the function of novel compounds in public MS/MS databases and to determine whether they are biochemical intermediates or true end products of natural product production.

In summary, this studied shows how MS/MS search algorithms can be used to mine publicly available data to determine the molecular diversity and ubiquity of specialized metabolites. The intensely studied specialized metabolites from *P. aeruginosa* that are known to play a role in virulence in humans are also found in environmental samples and in a large spectrum of molecular diversity that may expand their biological roles. One of the most promising aspects of readily available public MS/MS data is the ability to mine it for potential natural products of human interest, including modified forms of siderophores that had not previously been described. This approach represents a new tool in the belt of natural product chemists, microbial ecologists, and mass spectrometrists to further describe our knowledge of microbial chemical diversity.

## 4. Materials and Methods

### 4.1. P. aeruginosa Strains and Culture Conditions

Eight different clinical isolates of the bacterium were used for initial profiling of metabolite diversity (Table S1). These isolates were chosen based on being isolated from patients with different stages of CF and having both mucoid and non-mucoid phenotypes, to increase the potential of variation in metabolite production. These clinical isolates were cultured in Lysogeny Broth (LB) overnight at 37 °C with aerobic shaking of the culture.

### 4.2. Metabolite Extraction

All extractions were performed in a 2 mL-96 well deep-well plate. There were three different extraction methods tested in an attempt to increase the diversity of *P. aeruginosa* metabolites detected. The first method used a two-step extraction as described in [6]. First, ethyl acetate was added to the liquid culture in a 2:1 ratio, samples were vortexed to mix and allowed to incubate at room temperature for one hour. After incubation resulting in a clear separation of two liquid phases, the ethyl acetate phase was removed and placed into a second deep well 96 well plate (2 mL volume per well). Cold methanol was then added to the remaining bacterial sample in a ratio of 4:3 cold methanol to sample, vortexed and placed in and 4 °C refrigerator overnight. This extract was then spun down and added to the ethyl acetate extract for mass spectrometry analysis. The second extraction method tested was simply the addition of cold methanol to clinical isolate cultures in a 2:1 ratio. These samples were then vortexed for 20 s, incubated overnight in at 4 °C and then spun at 10,000× *g* for 30 s to pellet debris. The methanol layer remaining after centrifugation was used for mass spectrometry analysis. The third method used room temperature ethanol in a ratio of 2:1 ethanol to sample. These samples were also incubated in a 4 °C refrigerator overnight, vortexed and spun down as described above.

### 4.3. LC-MS/MS Mass Spectrometry

Metabolite extracts from all eight cultures were analyzed on a Thermo^®^ QExactive^®^ (Thermo Electron North America, Madison, WI, USA) and Vanquish Flex Binary UHPLC LC-MS/MS system (Thermo Electron North America, Madison, WI, USA) according to the methods of [29]. Briefly, the mobile phase was 0.1% formic acid in Milli-Q water and acetonitrile. The stationary phase was an Acquity^®^ reverse phase UPLC BEH C-18 column (2.1 mm × 100 mm, Waters^®^, Wood Dale, IL, USA). The chromatographic run was a total of 12 min-long as follows: 0–1 min 2% B, 1–8 min 2–100% B, with MS/MS data acquired for the first 10 min. This 100% B solution was then held for 2 min followed by a switch to 2% B for the remaining 2 min. The injection volume was 10 µL, the flow rate 0.40 mL/min and the column temperature 60 °C. Full MS^1^ mass spectra were collected in positive mode with a scan range set from *m*/*z* 100 to 1500 for the full MS mode (minutes 1–10 of run) at 35,000 resolution. Data dependent MS/MS parameters were a 17,500 resolution, automatic gain control (AGC) target of 1 × 10^5^, and a stepped fragmentation of 20, 40 and 60 normalized collision energy (NCE). The apex trigger was set from 0.1 to 2.5 s, an intensity threshold of 1 × 10^5^, isotope exclusion was on and dynamic exclusion was set to 3 s. Raw files (.raw) were converted to the .mzXML format for analysis.

### 4.4. Molecular Networking and MASST Searching

Molecular networks were generated for the metabolomics data from all three extracts from each clinical isolate with two different approaches. For molecular discovery of *P. aeruginosa* metabolite diversity, classic molecular networking was used [3]. Networks were built on GNPS using the classic molecular networking workflow [3]. Networking parameters were as follows: precursor mass tolerance 0.02 Da, fragment mass tolerance 0.02 Da, minimum cosine 0.65, minimum matched peaks of 4, and minimum cluster size of two. Library searching used the same parameters. The network used for data analysis is available here: https://gnps.ucsd.edu/ProteoSAFe/status.jsp?task=e86f338bc1764dbc979140e541d21bec. Molecular networks were analyzed for the presence of quinolones, rhamnolipids, phenazines and pyochelin. Each node in the network represents a clustered MS/MS spectrum according to [3] and contains an associated parent mass and retention time. These values were manually inspected for any duplication in the network by assessing a parent mass tolerance of less than 0.02 and a retention time tolerance of less than 7 s. Two nodes that fell within these ranges were considered duplicates and only one representative node was used for molecular descriptions. Nodes with mixed MS/MS patterns of one or more of the three quinolone types were also removed.

For MASST searching, the Feature Based Molecular Networking approach was used to avoid MS noise drifting into the MASST searches. The FBMN job is available here: https://gnps.ucsd.edu/ProteoSAFe/status.jsp?task=bbc0cca9ce7548a592cfc676147bd9d0. Furthermore, nodes selected for MASST searching were manually verified to have MS/MS patterns matching known quinolones, rhamnolipids or phenazines to avoid drifting away from these molecular families. Once a node was selected, the automatic MASST search button available for each node in a network was selected to run the algorithm against the entire GNPS database [4]. MASST search parameters were as follows: parent mass tolerance of 0.02, a minimum of 4 matched peaks and a cosine threshold of 0.7. After the search was completed, the dataset hits were recorded and the metadata attached to each dataset was mined for its source information including sample type and organism, if available. MASST search results for the pyochelin secondary network is found here: https://gnps.ucsd.edu/ProteoSAFe/result.jsp?view=network_displayer&componentindex=485&highlight_node=1052187&task=6660391aae4a4d789880c77c9b185835#%7B%7D. The *P. aeruginosa* metabolomics data are publicly available on the GNPS MassIVE server under project ID#MSV000086107.

## Supplementary Materials

The following are available online at https://www.mdpi.com/2218-1989/10/11/445/s1, Figure S1: Quinolone fragmentation, Figure S2: Quinolones in GNPS, Figure S3: Pyochelin chromatograms, Figure S4: Pyochelin fragmentation, Table S1: Strains used in experiment.
