# Supplementary material for: Mining Public Mass Spectrometry Data to Characterize the Diversity and Ubiquity of P. aeruginosa Specialized Metabolites
Source: Metabolites. 2020 Nov 5;10(11):445. doi: 10.3390/metabo10110445 (PMC7694397; doi:10.3390/metabo10110445)
Supplement: Supplementary file 1 [file metabolites-10-00445-s001.zip › metabolites-943342-supplementary-conversion/metabolites-943342-supplementary-revised-1-done.docx]

Mining Public Mass Spectrometry Data to Characterize the Diversity and Ubiquity of *P. aeruginosa* Specialized Metabolites

Andrew Lybbert^1^, Justin L. Williams^1,2^, Ruma Raghuvanshi^1^, A. Daniel Jones and Robert A. Quinn^1^

^1^Department of Biochemistry and Molecular Biology, Michigan State University, East Lansing, MI, USA

^2^University of Arkansas at Pine Bluff, Pine Bluff, AR.

**Supplementary Material**

**Figure S1.** Structures and MS/MS patterns of HHQ quinolones described in this study highlighting the unique fragment produced from those with an unsaturated bond between the first and second carbon of the fatty acid chain.

**Figure S2.** Number of studies where each quinolone was detected by MASST searching in GNPS according to the parent mass.

**Figure S3.** Extracted ion chromatogram of pyochelin and its related metabolites found in the *P. aeruginosa* clinical isolate data.

Figure S4. MS/MS fragmentation patterns of pyochelin and its novel related metabolites from the *P. aeruginosa data*. Putative structures of fragments are shown and the novel chemistry is highlighted. Note that the location of the hydroxyl group in red cannot be definitively determined with our data.

**Figure S5.** Arrow directed fragmentation patterns of predicted fragment structures from pyochelin MS/MS spectra.

**Figure S6.** Arrow directed fragmentation patterns of predicted fragment structures from Hydroxypyochelin MS/MS spectra.

**Figure S7.** Arrow directed fragmentation patterns of predicted fragment structures from Dehydroxypyochelin MS/MS spectra.

**Figure S8.** Arrow directed fragmentation patterns of predicted fragment structures from pyochelin amide MS/MS spectra.
